# Growth factor independent-1 Maintains Notch1-Dependent Transcriptional Programming of Lymphoid Precursors

**DOI:** 10.1371/journal.pgen.1003713

**Published:** 2013-09-12

**Authors:** James D. Phelan, Ingrid Saba, Hui Zeng, Christian Kosan, Malynda S. Messer, H. Andre Olsson, Jennifer Fraszczak, David A. Hildeman, Bruce J. Aronow, Tarik Möröy, H. Leighton Grimes

**Affiliations:** 1 Division of Cellular and Molecular Immunology; Cincinnati Children's Hospital Medical Center, Cincinnati, Ohio, United States of America; 2 Institut de recherches cliniques de Montréal IRCM, Montréal, Québec, Canada; 3 Département de microbiologie, infectiologie et immunologie, Université de Montréal, Montréal, Québec, Canada; 4 Institute of Infectious Diseases, Beijing Ditan Hospital, Capital Medical University, Beijing, China; 5 Division of Biomedical Informatics, Cincinnati Children's Hospital Medical Center, Cincinnati, Ohio, United States of America; 6 Division of Experimental Hematology; Cincinnati Children's Hospital Medical Center, Cincinnati, Ohio, United States of America; Baylor College of Medicine, United States of America

## Abstract

Growth factor independent 1 (Gfi1) is a transcriptional repressor originally identified as a gene activated in T-cell leukemias induced by Moloney-murine-leukemia virus infection. Notch1 is a transmembrane receptor that is frequently mutated in human T-cell acute lymphoblastic leukemia (T-ALL). Gfi1 is an important factor in the initiation and maintenance of lymphoid leukemias and its deficiency significantly impedes Notch dependent initiation of T-ALL in animal models. Here, we show that immature hematopoietic cells require Gfi1 to competently integrate Notch-activated signaling. Notch1 activation coupled with Gfi1 deficiency early in T-lineage specification leads to a dramatic loss of T-cells, whereas activation in later stages leaves development unaffected. In Gfi1 deficient multipotent precursors, Notch activation induces lethality and is cell autonomous. Further, without Gfi1, multipotent progenitors do not maintain Notch1-activated global expression profiles typical for T-lineage precursors. In agreement with this, we find that both lymphoid-primed multipotent progenitors (LMPP) and early T lineage progenitors (ETP) do not properly form or function in *Gfi1^−/−^* mice. These defects correlate with an inability of *Gfi1^−/−^* progenitors to activate lymphoid genes, including *IL7R*, *Rag1*, *Flt3* and *Notch1*. Our data indicate that Gfi1 is required for hematopoietic precursors to withstand Notch1 activation and to maintain Notch1 dependent transcriptional programming to determine early T-lymphoid lineage identity.

## Author Summary

Understanding the mechanisms that protect lymphoid cells from transformation is a critical first step in developing therapies against blood cancers. Recently, we demonstrated that the Growth factor independent-1 transcriptional repressor protein is required for cancer development driven by activation of Notch1 signaling. Here, we investigated the mechanisms by which Gfi1 protects lymphoid transformation. Using complex genetic mouse models to delete Gfi1 and activate Notch1, we demonstrate that Gfi1 is required to maintain both the homeostatic levels of Notch1 target genes in normal lymphoid precursors in the bone marrow, as well as to maintain the supraphysiologic levels of Notch1 signaling present in pre-malignant lymphoid progenitors. Consequently, without Gfi1 the pool of premalignant cells available for transformation is depleted. Our data provide additional insight into the multiple mechanisms by which developmental networks may have evolved to protect lymphoid cells from transformation**.**

## Introduction

Growth factor independent-1 (Gfi1) is a transcriptional repressor originally identified as a common proviral insertion site of the murine Moloney leukemia virus (MMLV) that conferred IL-2 independent growth to IL-2 dependent T-cell lymphomas [1]. Subsequently, *Gfi1* was identified as the most commonly activated gene in MMLV-induced lymphoid malignancies [2]. Gfi1 contains an N-terminal “SNAG” domain that is required for transcriptional repression and nuclear localization [3] and six zinc fingers of which, three, four and five are required for specific DNA-binding [4], [5]. *Gfi1^−/−^* mice display decreased HSC fitness, an accumulation of myeloid progenitors, and a lack of mature neutrophils [6], [7], [8]. Furthermore, germline deletion of *Gfi1* results in a 4-fold decrease in thymic cellularity and modest increases in apoptotic cells [9]; whereas, mice with a *CD4*-promoter-driven Cre and floxed *Gfi1* alleles (*Gfi1^f/f^*) demonstrate no defects in absolute thymocytes numbers[10]. Taken together, these data have been interpreted to mean that *Gfi1^−/−^* thymic phenotypes are largely due to Gfi1 anti-apoptotic functions during early thymopoiesis.

Notch1 is a transmembrane receptor that is critical throughout metazoan development acting as a molecular switch to determine cell fate. Similarly, during hematopoiesis, activation of Notch1 is required for proper T cell development [11], [12], [13], [14], [15]. T cells arise from circulating bone marrow progenitors that enter the thymus and encounter Notch1 ligands of the Delta-like and Jagged family [16], [17], [18]. Ligand-engagement of Notch receptors results in a conformational change exposing internal cleavage sites. A disintegrin and metalloprotease (ADAM)- and γ-secretase complex-mediated cleavage results in intracellular Notch (ICN) release from the membrane, nuclear translocation [19], [20], [21], and subsequent binding to CBF1/Suppressor of Hairless/Lag1 (CSL/Rbpj-κ) ultimately leading to Notch target gene activation. As Notch1 signal strength increases in early T lineage progenitors (ETP) through double negative (DN) 3 pro-T cells, transcriptional programs are upregulated which enforce T lymphoid identity at the expense of other lineages [22]. Notch1 signaling strength is highest leading up to TCRβ-selection, however, early progenitors in the BM may also require low level Notch signals as one component of the stimulus to proliferate and differentiate into lymphoid progenitors. Although Notch1 signaling may not be required for the maintenance of adult hematopoietic stem cells [23], [24], it functions as a tumor suppressor during myeloid development [25], and inhibition of Notch1 in progenitors dramatically reduces the formation of ETPs disrupting downstream stages of T-cell development in the thymus [26].

T cell acute lymphoblastic leukemia (T-ALL) is a subset of acute lymphoblastic leukemia, the most prevalent pediatric malignancy comprising nearly 25% of all childhood cancers [27]. Translocations placing *NOTCH1* under control of the *TCRb* locus, t(7;9)(q34;q34.3) first implicated *NOTCH1* in T-ALL [28]. Yet additional activating *NOTCH1* mutations were found in more than 50% of T-ALL patients [29]. Moreover, mutations in *NOTCH1* [30] and *NOTCH1* regulatory proteins [31] have also been identified in T-ALL [32] . All of these mutations are thought to create constitutively active forms of ICN through ligand-independent activation and ICN nuclear translocation [33]. Mutations in *GFI1* have not been detected in human T-ALL [34] [32]; however, transgenic overexpression of Gfi1 can accelerate oncogene-driven murine models of T-ALL [35], [36].

Recently, we identified Gfi1 as an important factor in the initiation and maintenance of lymphoid leukemias [37]. Interestingly, in human T-ALL patients with *NOTCH1* mutations, or a transcriptional signature indicative of activated NOTCH1, *GFI1* was highly expressed; while in mice, Gfi1 loss of function profoundly blocked Notch-initiated leukemia. To further investigate this unique relationship, we used genetic mouse models, which constitutively and inducibly delete *Gfi1*, to demonstrate that *Gfi1* is required in a cell autonomous manner for early thymocytes and lymphoid progenitors in the bone marrow to competently receive Notch signals. Furthermore, we show that *Gfi1* ^−/−^ lymphoid progenitors cannot respond to endogenous levels of Notch1, potentially explaining the dramatic reduction in *Gfi1* ^−/−^ ETP and LMPP numbers. Thus, our findings identify Gfi1 as a critical factor in the response of immature hematopoietic cells to Notch1 signaling.

## Results

### Loss of *Gfi1* and activation of intracellular Notch1 results in thymic hypoplasia

**Figure 1:**
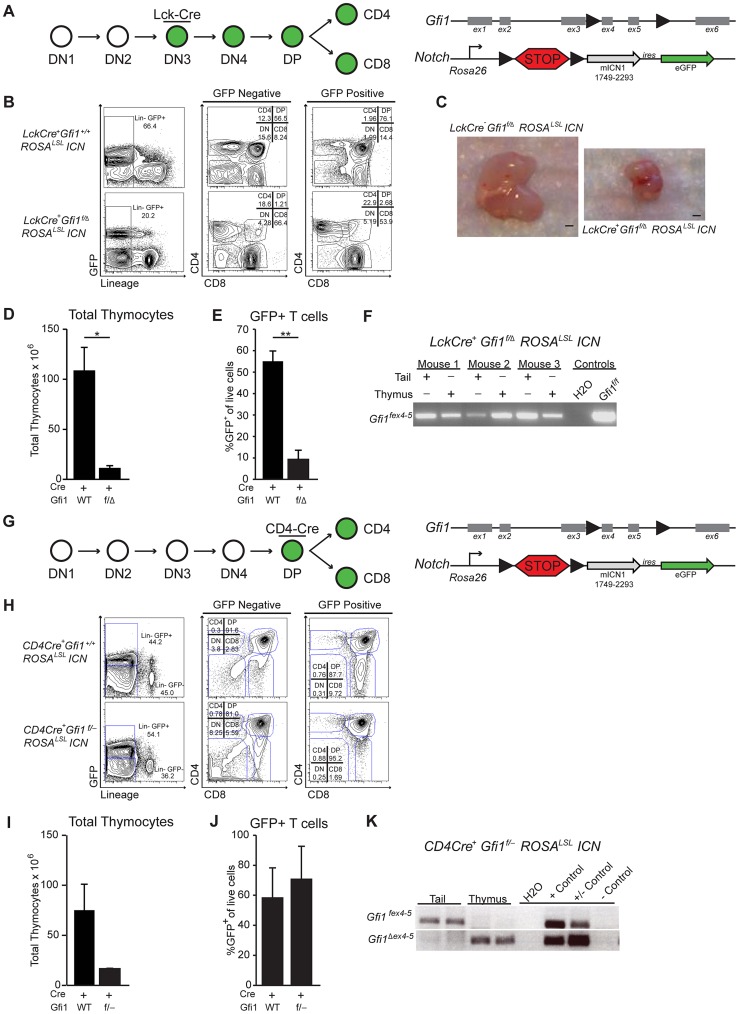
Loss of *Gfi1* and activation of intracellular Notch1 results in thymic hypoplasia. (A) Top left: Schematic of T cell development demonstrating that the proximal Lck-driven Cre activity is off during early stages (DN1-2) but is activated later during the DN3 stage of T cell development. Top right: Schematic of the floxed *Gfi1* locus as well as the ICN transgene which has a floxed “STOP” cassette (lsl) preventing activation of ICN and GFP. (B) Example plots of flow cytometric analysis of thymic T cell populations for both GFP negative (left) and GFP positive (right) cells from *LckCre^+^Gfi1^+/+^ROSA^LSL^ICN* and *LckCre^+^Gfi1^f/Δ^ROSA^LSL^ICN* mice. (C) Photograph of thymi from indicated mice. Scale bar is 1 mm. (D) Total thymocyte numbers from *LckCre^+^ Gfi1^+/+^ROSA^LSL^ICN* (n = 20) and *LckCre^+^Gfi1^f/Δ^ROSA^LSL^ICN* (n = 4) mice. (E) Percentage of live, eGFP-expressing (a marker of Notch activation) thymocytes as determined by flow cytometric analysis. (F) PCR analysis of 3 separate mice for floxed alleles of *Gfi1* (*Gfi1^fex4–5^*). (G) Top left: Schematic of T cell development demonstrating the CD4-driven Cre activity is off in early stages (DN1–4) but activates later during the DP stage of T cell development. Top right: Schematic of the floxed *Gfi1* locus as well as the ICN transgene which has a floxed “STOP” cassette (lsl) preventing activation of ICN and eGFP. (H) Example plots of flow cytometric analysis of thymic T cell populations for both GFP negative (left) and GFP positive (right) cells from *CD4Cre^+^Gfi1^+/+^ROSA^LSL^ICN* and *CD4Cre^+^Gfi1^f/−^ ROSA^LSL^ICN* mice. (I) Total thymocyte numbers from *CD4Cre^+^Gfi1^+/+^ROSA^LSL^ICN* (n = 9) and *CD4Cre^+^Gfi1^f/−^ ROSA^LSL^ICN* (n = 2) mice. (J) Percentage of live, eGFP-expressing (a marker of Notch) thymocytes as determined by flow cytometric analysis. (K) PCR analysis of two mice for floxed alleles (top) or deleted alleles of *Gfi1* (bottom). Representative FACS plots and pictures are shown. Experiments were repeated 2–3 times. Averages with SEM are shown in bar graphs. Students T-test were performed, *p≤0.05, **p≤0.01.

To further elucidate the mechanisms that protect *Gfi1* deficient T cells from T-ALL transformation, we investigated the requirement for Gfi1 in developing T cells exposed to Notch1 activation. To do so, we bred mice in which *Cre* recombinase expression is driven by the T-cell-specific proximal-*Lck* promoter [38] with both *Gfi1^fex4–5^* (*Gfi1^f^*) mice and germline *Gfi1^Δex2–3^* (*Gfi1^−^*) or *Gfi1 ^Δex4–5^* deficient mice (*Gfi1 ^Δ^*) resulting in *LckCre^+^Gfi1^f/−^* (or *LckCre^+^Gfi1^f/Δ^)* animals. Notably, we observed a similar 3–4-fold reduction in total thymocytes as previously published in *Gfi1* germline deleted mice [9] (Figure S1). Next, we bred the *LckCre^+^Gfi1^f/Δ^* model with a *Rosa26*-driven intracellular-Notch1 (ICN) transgene, in which ICN-IRES-eGFP expression is prevented by a floxed “stop” cassette (*ROSA^lsl^ICN*) [39]. In the *LckCre^+^Gfi1^f/Δ^ ROSA^lsl^ICN* mice, Cre expression should activate ICN and eGFP expression while simultaneously deleting *Gfi1* (Figure 1A). As previously reported [40], we find that ICN activation, in the presence of Gfi1, leads to an accumulation of DP and CD8^+^ T cells at the expense of CD4^+^ cells (Figure 1B, GFP Positive *LckCre^+^Gfi1^+/+^ROSA^lsl^ICN*). In contrast, when activation of ICN is coupled with *Gfi1* deletion, the majority of GFP^+^ cells are CD4 or CD8 single positive cells (Figure 1B, GFP positive, *LckCre^+^Gfi1^f/Δ^ ROSA^lsl^ICN*). Moreover, ICN expression coupled with *Gfi1* deletion led to a dramatic reduction in thymus size (Figure 1C). Further analysis of total thymocyte numbers revealed a 17-fold decrease in total cellularity when activation of ICN was combined with loss of *Gfi1* (Figure 1D, p<0.05). Notably, this phenotype was not observed in control *LckCre^+^ROSA^lsl^ICN* or in *LckCre^+^Gfi1^f/Δ^* thymocytes where activation of ICN or deletion of *Gfi1* occurs separately (Figure 1D and Figure S1). The few remaining thymocytes present in the *LckCre^+^Gfi1^f/Δ^ ROSA^lsl^ICN* mice either lacked equivalent ICN transgene activation, as measured by eGFP (Figure 1E, p<0.01,) or failed to delete the floxed allele of *Gfi1* (Figure 1F). Moreover, the significant decrease in the percentage of GFP^+^ cells in *LckCre^+^Gfi1^f/Δ^ ROSA^lsl^ICN* mice (Figure 1E) is underrepresented by the flow cytometric plots shown. For example, the absolute number of GFP^+^ thymocytes in *LckCre^+^ROSA^lsl^ICN* mice is 49.8×10^6^ versus 0.35×10^6^ GFP^+^ thymocytes in *LckCre^+^Gfi1^f/Δ^ ROSA^lsl^ICN* mice, a 142-fold decrease in the total number of GFP^+^ thymocytes between ICN-signaled Gfi1-sufficient versus ICN-signaled Gfi1-deficient cells. Taken together, these data demonstrate that Gfi1 is required to withstand chronic ICN signaling during the stages of development in which T cell malignant transformation occurs [41], [42].

To determine whether this apparent synthetic lethal relationship was dependent upon the stage of transgene activation or whether Notch-signaled pre-leukemic T cells generally require Gfi1, we utilized CD4Cre transgenic mice and repeated the above experiments. Notably, CD4Cre is expressed in DP thymocytes, and deletion of floxed *Gfi1*, *Notch1*, or *Rbpj-κ* by CD4Cre does not result in a reduction of thymocytes [10], [43], [44]. Therefore, any lethality caused by deleting Gfi1 and activating Notch should not be due to a specific developmental requirement for these factors alone, but instead would reflect a synergistic phenotype. Thus, we bred CD4Cre transgenic mice to *Gfi1^f/−^ ROSA^lsl^ICN* mice (Figure 1G) and examined the effects on thymocyte development. Similar to LckCre-mediated activation, CD4Cre activation of ICN lead to an accumulation of DP and CD8 SP T cells at the expense of other populations (Figure 1H, *CD4Cre^+^ROSA^lsl^ICN*, GFP Positive). Comparatively, deletion of *Gfi1* led solely to the development of DP T cells (Figure 1H, *CD4Cre^+^ Gfi1^f/−^ ROSA^lsl^ICN*, GFP Positive). However, in contrast to the published *CD4CreGfi1^fex4–5/fex4–5^* mice [10] or *CD4Cre^+^ROSA^lsl^ICN mice*, the *CD4Cre^+^Gfi1^f/−^ ROSA^lsl^ICN* mice displayed a dramatic decrease in total cellularity similar to *LckCre^+^Gfi1^f/−^ROSA^lsl^ICN* mice (Figure 1I). Despite the decrease in total number of thymocytes, the percentage of *CD4CreGfi1^f/−^ROSA^lsl^ICN* thymocytes able to activate the ICN transgene was equivalent in *CD4CreROSA^lsl^ICN* signaled cells with or without *Gfi1* as measured by eGFP (Figure 1J). Furthermore, *CD4CreGfi1^f/−^ ROSA^lsl^ICN* thymocytes were able to efficiently delete the floxed allele of *Gfi1* in thymocytes where Cre is active, but not in control Cre inactive tail tissue (Figure 1K). Thus, the presence of eGFP-expressing *Gfi1^Δ/−^* cells in this model suggests that the DP and SP T-cells do not absolutely require Gfi1 to express activated ICN, even though this combination results in dramatically decreased thymic cellularity.

### Peripheral T cells do not require Gfi1 to survive activated Notch signaling

**Figure 2:**
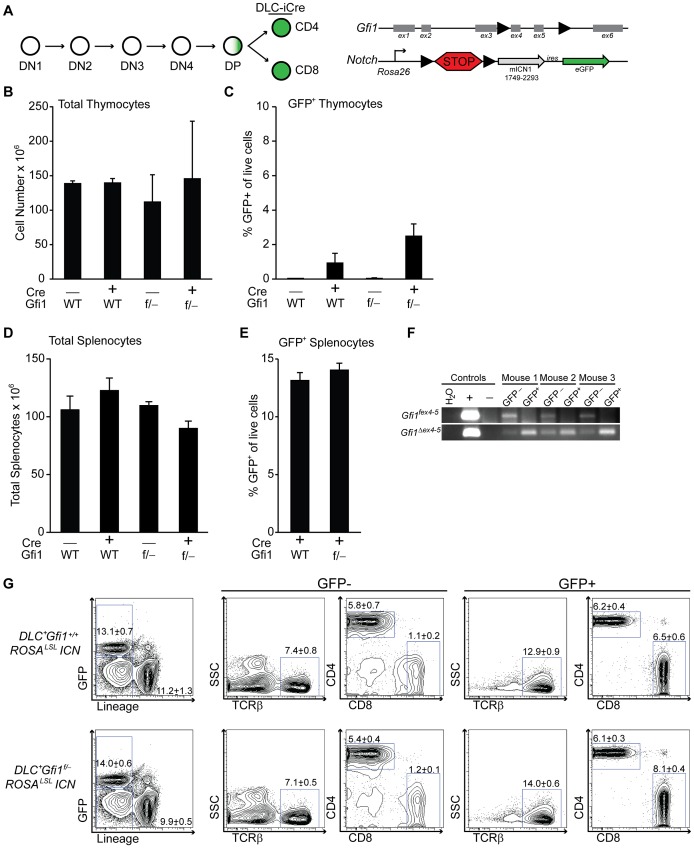
Peripheral T cells do not require Gfi1 for Notch activation. (A) Top left: Schematic of T cell development demonstrating the distal Lck-driven Cre (DLC-iCre) activity is off during most stages of T cell development, but activates in single-positive cells. Top right: Schematic of the floxed *Gfi1* locus as well as the ICN transgene which has a floxed “STOP” cassette (lsl) preventing activation of ICN and GFP. (B–C) Total thymocyte numbers (B) and percentage of live, GFP-expressing thymocytes (C) as determined by flow cytometric analysis of *Gfi1^+/+^ ROSA^LSL^ICN* (n = 4), *DLC-iCre^+^Gfi1^+/+^ROSA^LSL^ICN* (n = 2), *Gfi1^f/−^ROSA^LSL^ICN* (n = 2), and *DLC-iCre^+^Gfi1^f/−^ROSA^LSL^ICN* (n = 4) mice. Averages with SD are shown (B–C). (D) Total splenocyte numbers of *Gfi1^+/+^ ROSA^LSL^ICN* (n = 8), *DLC-iCre^+^Gfi1^+/+^ROSA^LSL^ICN* (n = 10), *Gfi1^f/−^ROSA^LSL^ICN* (n = 5), and *DLC-iCre^+^Gfi1^f/−^ROSA^LSL^ICN* (n = 16) mice. (E) Percentage of live, GFP-expressing splenocytes as determined by flow cytometric analysis. (F) PCR analysis of splenic T cells from three *DLC-iCre^+^Gfi1^f/−^ROSA^LSL^ICN* mice independently FACS-sorted for TCRβ^+^ GFP^−^ or GFP^+^ cells and genotyped for floxed (top) and deleted (bottom) alleles of *Gfi1*. (G) Example plots of flow cytometric analysis of splenic T cell populations for both GFP negative (left) and GFP positive (right) cells. Representative FACS plots and pictures are shown. Experiments were repeated 2–3 times. Averages with SEM are shown in bar graphs (D–E). One-way ANOVAs and student T tests were performed but no significant differences were found.

To more precisely define the developmental stages susceptible to ICN activation and *Gfi1* deletion, we mated the *ROSA^lsl^ICN* or *Gfi1^f/−^ ROSA^lsl^ICN* transgenic mice to transgenic mice that activate *Cre* expression after TCR positive selection (*distal-LckCre = DLC*) [45]. Similar to published reports, we found that less than 5% of the thymocytes in *DLC^+^ROSA^lsl^ICN* or *DLC^+^Gfi1^f/−^ ROSA^lsl^ICN* expressed eGFP, and only at very late stages of T cell development (Figures 2A–C, S2). As such, we examined peripheral splenic T cells and found no statistical differences in total cellularity (Figure 2D) or in the percentages of GFP^+^ T cells between *DLC^+^ROSA^lsl^ICN* and *DLC^+^Gfi1^f/−^ROSA^lsl^ICN* mice (Figure 2E). Furthermore, FACS sorted GFP^+^ T cells displayed complete excision of the floxed allele of *Gfi1* (*Gfi1^fex4–5^*) and had detectable levels of the deleted allele of *Gfi1* (*Gfi1^Δ ex4–5^*, Figure 2F). These cells still demonstrated a partial phenocopy of *Gfi1* deficiency in that they have an increase in the frequency of the CD8^+^ population (Figure 2G); however, no differences were observed in the immunophenotype of ICN-activated T cells, with or without *Gfi1*. These data provide strong evidence to suggest that the ICN^+^ *Gfi1^Δ^* ^/*Δ*^ -induced hypocellularity phenotype is limited to a window during development in which T cells are susceptible to transformation (i.e. after TCRβ-selection). However, as that window closes and developmental transcriptional programs turn off, they are no longer susceptible to phenotypes caused by ICN activation and *Gfi1* deletion.

### Gfi1 is required for lymphoid lineage priming

Having established that deletion of *Gfi1* early in T cell development mimics the phenotype of *Gfi1* germline deletion, (*LckCre^+^Gfi1^f/Δ^*, Figure S1A–E) and that overexpression of intracellular Notch1 does not rescue this defect (*LckCre^+^Gfi1^f/Δ^ ROSA^lsl^ICN*, Figure 1A–F) we further observed a direct relationship between the stage of lymphoid developmental and the synthetic lethal combination of deleting *Gfi1* and activating ICN. This combination was most profound in early stages of T cell development (LckCre) in that GFP+ *Gfi1^Δ^* ^/*Δ*^ cells were not detectable. In contrast, at later developmental stages (CD4Cre) the absolute requirement for Gfi1 was lost (albeit with hypocellularity) and GFP+ *Gfi1 ^Δ^* ^/*Δ*^ T cells could be detected. However, at very late stages of T cell development (DLCre) GFP+ *Gfi1^Δ^* ^/*Δ*^ T cells could be detected with no obvious defect in the numbers of peripheral or thymic T cells. Thus, we hypothesized that Gfi1 must be most critical during the earliest stages of lymphoid development where progenitors first experience lymphoid transcriptional programming, which includes Notch1 signaling. However, these data do not delineate between a selective event in which cells without Gfi1 die, versus an instructive event in which cells without Gfi1 fail to undergo proper lineage commitment and lymphoid gene expression changes.

**Figure 3:**
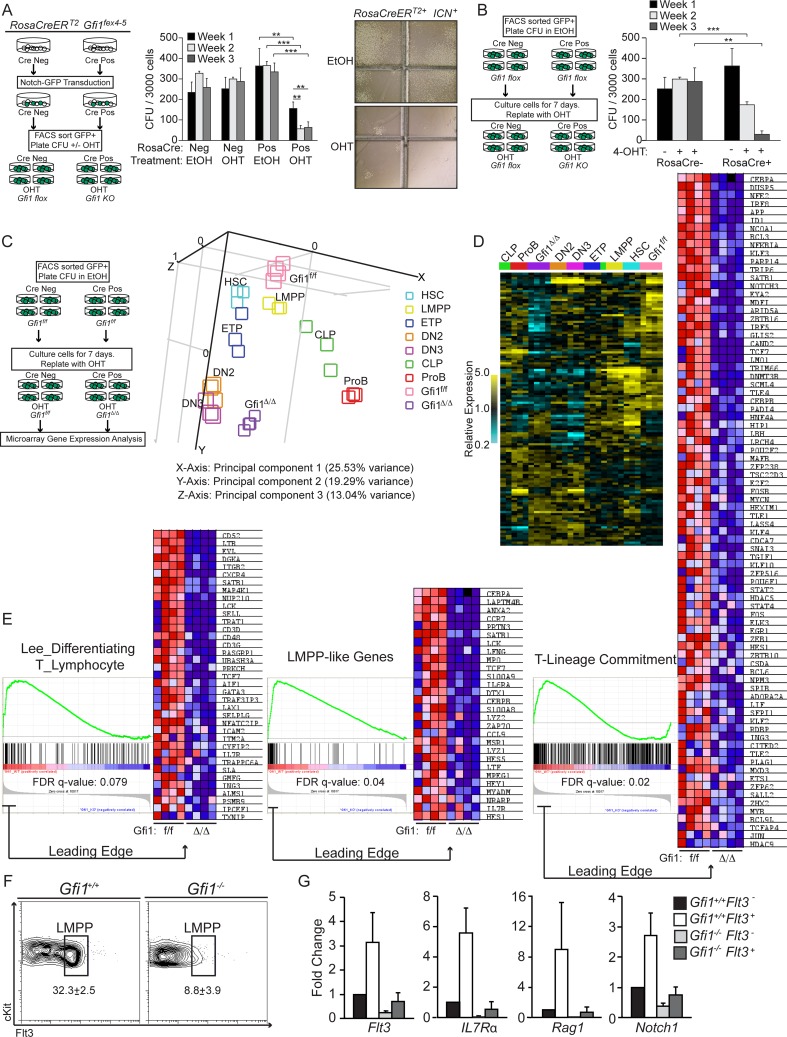
Gfi1 is required to enforce ICN1 activation of lymphoid genes. (A) Schematic for ICN colony forming unit (CFU) assay +/− Gfi1. CFU from FACS sorted ICN^+^eGFP expressing Lin− cells from either *Gfi1^f/f^* or *RosaCreER^T2+^ Gfi1^f/f^* were plated at 3000 cells/plate in 1 µM 4-OHT or EtOH control and cultured for 7 days (left). CFU/plate were then enumerated, disrupted and replated at 3000 cells/plate for additional two platings (middle). Representative pictures of *RosaCreER^T2+^Gfi1^f/f^* in EtOH or 4-OHT are shown (right). (B) Schematic of FACS-sorted ICN^+^eGFP expressing Lin− cells from either *Gfi1^f/f^* or *RosaCreER^T2+^Gfi1^f/f^* plated at 3000 cells/plate in EtOH and cultured for 7 days, then replated in 1 µM 4-OHT for an additional two platings (left). Enumeration of CFU (right). (C) Schematic of CFU conditions: similar to (B), but after 7 days in 1 µM 4-OHT, CFU were disrupted and RNA was isolated for microarray analysis (left). Principal component analysis of CFU with FACS-sorted HSC and lymphoid progenitor populations is shown. Note that *Gfi1^f/f^* CFU, but not *Gfi1^Δ/Δ^* CFU, cluster with LMPP. (D) Heatmap of 125 statistically different T-lineage commitment genes between *Gfi1^f/f^* CFU and *Gfi1^Δ/Δ^* CFU in indicated HSC and progenitor populations. Note that although *Gfi1^Δ/Δ^* CFU cluster with DN2 & DN3 cells, many of the 125 genes show differential expression suggesting *Gfi1 ^Δ/Δ^* CFU are not characteristic of normal DN2 or DN3 cells. (E) GSEA enrichment plots and leading edge heatmaps demonstrate *Gfi1^f/f^* CFU transcriptionally mimic the expression of the indicated genesets, specific for lymphoid progenitors, while *Gfi1^Δ/Δ^* CFU fail to induce these genes. (F) Flow cytometric plots of Flt3^+^ LSK (LMPP) in BM progenitors from *Gfi1^+/+^* and *Gfi1^−/−^* mice (N = 6/genotype); averages with SEM are shown. (G) Relative gene expression of indicated genes from FACS sorted Flt3^−^ and Flt3^+^ LSK from *Gfi1^+/+^* and *Gfi1^−/−^* mice. Averages with SD are displayed from triplicates (N = 2).

To clarify this, we next performed a series of *in vitro* assays to concisely test the cell autonomous requirement for Gfi1 in lymphoid priming by inducibly deleting *Gfi1* in the context of chronic ICN expression. First, we isolated Lin^−^ BM from *RosaCreER^T2^Gfi1^fex4–5^* and control *Gfi1^fex4–5^* mice, and retrovirally transduced the stem and progenitor cells with GFP-marked ICN. GFP^+^ cells were FACS-sorted and plated in methylcellulose as previously described [46], [47] in the presence of 4-hydroxy tamoxifen (4-OHT, to induce Cre activity and delete *Gfi1^fex4–5^*) or vehicle control (EtOH). After one week in culture, CFU were enumerated, methylcellulose was disrupted and CFU were re-plated into 4-OHT or control-containing methylcellulose. This process was repeated for three weeks of plating (Figure 3A, diagram left). Untransformed progenitor cells generally produce 100–200 CFU within the first week, but fail to produce robust CFU in subsequent replatings [5], [46], [47]. In the absence of Cre expression, 4-OHT had no effect on CFU number or replating ability (Figure 3A, middle, Cre Neg: EtOH vs. OHT). However, in the presence of Cre, 4-OHT treatment dramatically reduced the number of CFU (Fig. 3A middle, Week 1, Cre Pos: EtOH vs. OHT: 363 to 156, p<0.01). Replating of *Gfi1^f/f^*, or *RosaCre^+^Gfi1^f/f^* vehicle-treated CFU led to similar numbers of CFU seven days later, whereas replating of *RosaCre^+^Gfi1^Δ^* ^/*Δ*^ resulted in an additional three-fold reduction in total CFU (Figure 3A middle, Week 1 vs. 2: 156 to 57, p<0.01). Moreover, the CFU that did form in the absence of Gfi1 displayed substantially fewer cells per CFU demonstrating their inability to respond to ICN overexpression in the same manner as *Gfi1^f/f^* controls (Figure 3A, right).

In the absence of ICN overexpression, interruption of Gfi1 function promotes monocytic over granulocytic CFU formation [5]. Activation of ICN in myeloid lineages has recently been suggested to be lethal [25]. To avoid potential confounding factors of ICN activation in Gfi1-deficient myeloid progenitors, we next repeated the above assay (Figure 3A), but after FACS-sorting GFP^+^ ICN-transduced Lin^−^ cells, we plated them for one week in the absence of 4-OHT in order to promote lymphoid priming and differentiation by ICN overexpression. After seven days in culture, CFU were enumerated, disrupted and plated in 4-OHT containing methylcellulose for an additional seven days for two rounds of replating (Figure 3B, left). Lymphoid-primed *Gfi1^f/f^* CFU were again unaffected by addition of 4-OHT through subsequent replatings. Although cells from *RosaCre^+^Gfi1^f/f^* generated equivalent CFU to cells from *Gfi1^f/f^* mice while cultured without 4-OHT, upon addition of 4-OHT, these cells again demonstrated a significant reduction in total CFU and cells per CFU compared to *Gfi1^f/f^* controls (Figure 3B right, Week 2: 300 to 174, p<0.001). These data suggest that lymphoid-primed CFU also require Gfi1 to competently respond to ICN signaling.

To verify that this *in vitro* model truly reflects the characteristics of lymphoid progenitors, we repeated the experiment and examined global gene expression patterns. ICN-transduced *Gfi1^f/f^* and *RosaCre^+^Gfi1^Δ^* ^/*Δ*^ lineage-negative bone marrow cells were cultured for seven days without 4-OHT (to induce lymphoid-priming,) and then an additional seven days in 4-OHT (to induce deletion of *Gfi1^f/f^* alleles) before RNA was isolated and microarray expression analysis was performed (Figure 3C, left). Recently, global RNA-seq and ChIP-seq analyses defined a subset of genes that definitively distinguish FACS-sorted early lymphoid populations based upon their transcriptional networks [48]. Restricting our analysis to these genes, we first questioned whether they demonstrated statistically significant gene expression differences with or without *Gfi1*. Of the 378 tested, 125 genes displayed p-values <0.05 and were then used to cluster the expression signatures from both ICN-transduced CFU as well as normal FACS sorted lymphoid progenitors (Table S1) [49]. Principal component analysis (PCA) clustered *Gfi1^f/f^* CFU closest to LMPP populations demonstrating that the CFU partially mimic important transcriptional programs of *in vivo* lymphoid progenitors (Figure 3C, right). However, upon loss of *Gfi1*, PCA revealed that *Gfi1^Δ^* ^/*Δ*^ CFU no longer cluster with LMPP (Figure 3C–D), demonstrating a global inability to maintain lymphoid progenitor priming.

We next used an unbiased approach and applied gene set enrichment analysis (GSEA) [50] to our entire dataset. GSEA showed enrichment of published lymphoid progenitor signatures in *Gfi1^f/f^* CFU, whereas *Gfi1^Δ^* ^/*Δ*^ CFU showed no such enrichment (Figure 3E, “Lee_differentiating T_lymphocyte”). The same enrichment pattern was observed using more recently published LMPP-like and T-lineage commitment gene lists not yet curated in the MSigDB (Figure 3E “LMPP-like Genes” & “T-Lineage Commitment”). Indeed, further analysis [51] of gene expression differences between *Gfi1^f/f^* and *Gfi1^Δ^* ^/*Δ*^ ICN CFU, demonstrated significant changes in cell surface markers (Table S2, GO Cellular Component GO:0009986, p<2.49×10^−16^) and CD antigen genes (Table S3, HUGO Genenames.org, p<3.40×10^−30^). These data suggest that much (but not all) of the ICN-instructed lymphoid progenitor programs are dependent upon Gfi1. Taken together, we conclude that i) ICN-transduced *Gfi1^+/+^* CFU share critical transcriptional programs with lymphoid bone marrow progenitors; ii) loss of Gfi1 results in a subsequent loss of key elements of those ICN-regulated transcriptional networks necessary for proper lymphoid lineage identity; and iii) Gfi1 is required in ICN-signaled (FACS sorted) cells in a cell autonomous fashion.

Given the similarity of gene signatures between ICN-CFU and LMPP and the reliance of these cells *in vitro* on Gfi1, we questioned whether endogenous levels of Notch1 signaling experienced *in vivo* by lymphoid progenitor cells of *Gfi1^−/−^* mice may engender the same phenotypes identified in the transgenic and retroviral overexpression systems. To answer this question, we examined the LMPP (Flt3 high, Lin^−^, cKit^+^, Sca1^+^) in the BM reasoning that: i) LMPP are the first lymphoid progenitors to respond to Notch1 signaling [52], ii) ICN^+^ *Gfi1^f/f^* CFU clustered closest to FACS-sorted LMPP, and iii) differences in the expression of Flt3 have been reported in *Gfi1^−/−^* LSK [7], [8]. Similar to previous reports [7], [53], we observed a decrease in the percentage and total number of LMPPs in *Gfi1^−/−^* mice (Figure 3F). To determine whether *Gfi1^−/−^* phenotypic LMPP are functionally similar to wild type LMPP, we FACS sorted LSK and LMPP from *Gfi1^+/+^* and *Gfi1^−/−^* mice and tested for the induction of lymphoid signature genes coincident with Flt3 expression in LMPP. Whereas *Gfi1^+/+^* progenitors upregulated the expression of *Flt3*, *IL7R*, *Rag1* and *Notch1* 3–10 fold during the transition from Flt3^−^LSK to LMPP, *Gfi1^−/−^* progenitors did not induce these genes to the same degree (Figure 3G). Furthermore, we found lower expression of each of these genes in *Gfi1^−/−^* Flt3^−^LSK, suggesting that phenotypically normal *Gfi1^−/−^* progenitors have a functional defect in their ability to prime lymphoid transcriptional programs. Taken together, these data indicate that loss of Gfi1 in lymphoid-primed progenitors results in a cell-autonomous inability to maintain a lymphoid specific transcriptional program.

### *Gfi1* deficiency results in loss of early thymic progenitors (ETP)

**Figure 4:**
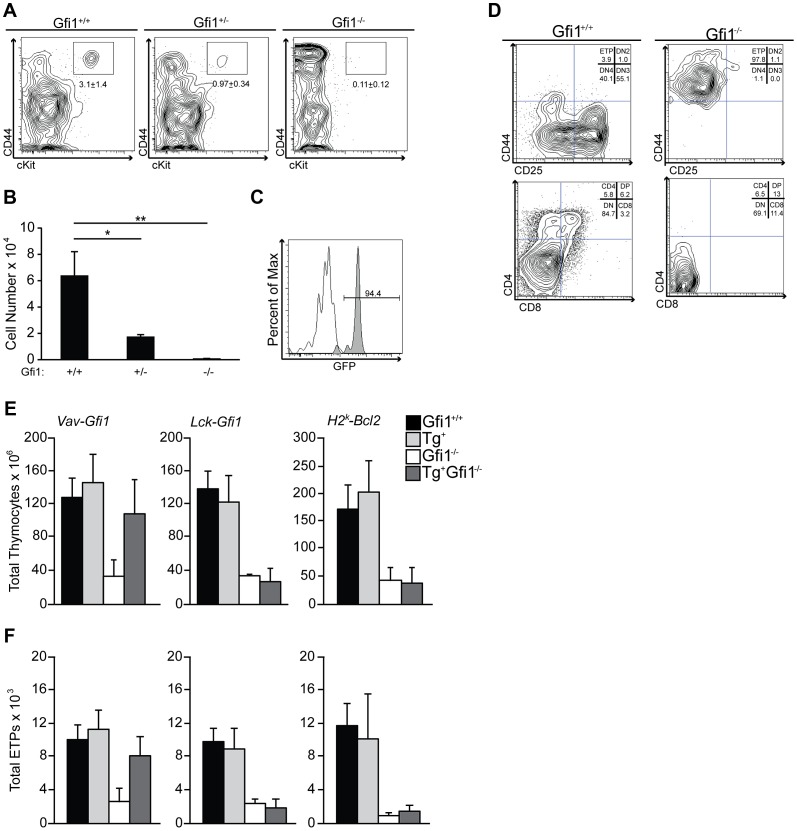
*Gfi1* deficiency results in reduced number and function of ETP. (A) *Gfi1^+/+^* (n = 4), *Gfi1^+/−^* (n = 6) and *Gfi1^−/−^* (n = 3) thymocytes were analyzed for the percent of Lin−, CD25−, cKit+, CD44+ ETPs; Average ± SD. (B) Total cell number or ETPs from (A). (C) Histogram of GFP from *Gfi1^GFP/+^* knock-in mice (grey shaded peak) compared to *Gfi1^+/+^* littermate control (white peak,) demonstrates Gfi1 expression in ETPs (N = 5/genotype). (D) ETPs were FACS-sorted from *Gfi1^+/+^* and *Gfi1^−/−^* mice directly onto OP9-DL1 stroma and cultured for 15 days. T cell development stages were assessed by flow cytometry. Experiments were repeated three times. One representative example is shown. (E–F) Transgenic rescue of *Gfi1^−/−^* T cell development using *Vav-Gfi1* (N = 9/genotype), *Lck-Gfi1* (N = 6/genotype), and *H2^K^-Bcl2* (N = 6/genotype) in *Gfi1^+/+^* and *Gfi1^−/−^* mice. Total thymocyte numbers (E) were calculated and flow cytometry was performed to identify ETPs (F).

ETPs are thought to be the progeny of lymphoid-primed progenitors, which reside within the BM and have significant overlap in transcriptional signatures with LMPP [49]. Therefore, we hypothesized that the role of Gfi1 during lymphoid priming may be most pronounced in ETPs, in particular since these cells experience a dramatic increase in Notch1 signaling. *Gfi1^+/−^* lymphoid progenitors express less Gfi1 protein than wild type cells [54], therefore we examined the effect of Gfi1 deletion and haploinsufficiency upon ETP numbers. We found a *Gfi1* dose-dependent reduction in ETP percentages and absolute numbers in *Gfi1* ^+/−^ and *Gfi1^−/−^* thymi compared to *Gfi1^+/+^* controls (Figure 4A–B; 6.4×10^4^, 1.7×10^4^, 0.06×10^4^ between *Gfi1^+/+^*, *Gfi1* ^+/−^ and *Gfi1^−/−^* respectively; p<0.05 and p<0.01). Thus, we conclude that *Gfi1^−/−^* mice have few phenotypically normal ETPs.

Next, we asked whether ETPs normally express Gfi1 and whether *Gfi1^−/−^* ETPs can respond to Notch1 signaling. To address the first question, we used *Gfi1-GFP* knock-in mice (*Gfi1^GFP/+^*) [55] in which eGFP replaces *Gfi1* coding exons, and the expression of eGFP mirrors that of endogenous *Gfi1.* We found that *Gfi1^GFP/+^* ETP are clearly eGFP^+^, demonstrating that *Gfi1* is highly expressed in ETPs (Figure 4C). To address the latter question, we FACS-sorted *Gfi1^+/+^* and *Gfi1^−/−^* ETP cells and exposed them to the Notch ligand, Delta-like 1 (DL1), by culturing the cells on OP9-DL1 stroma. *Gfi1^−/−^* ETP failed to respond and did not progress through T cell development whereas their *Gfi1^+/+^* ETP controls began to express both CD4 and CD8 after 15 days in culture (Figure 4D). Thus, these data demonstrate that phenotypically defined Gfi1 deficient ETPs do not properly function in response to Notch ligands *in vitro*.

We next sought to genetically rescue Gfi1 expression both before and after lymphoid progenitors experience increases in basal Notch1 signaling. To examine whether endogenous levels of Notch1 signaling were correctly interpreted, we examined total thymocyte and ETP numbers, both of which are critically dependent on Notch1 [11], [26]. First, we mated *Vav-Gfi1* transgenic mice, which express *Gfi1* in all hematopoietic stem/progenitors and mature lineages [56], to germline *Gfi1^−/−^* mice. Gfi1 expression in this model occurs before endogenous increases in Notch1 signals [56]. We then analyzed the total number of thymocytes and the formation of ETPs by flow cytometry. *Vav*-mediated expression of *Gfi1* rescued both the total thymocyte numbers (Figure 4E) and the total numbers of ETPs (Figure 4F) to the levels of *Gfi1^+/+^* controls. Next, we mated germline *Gfi1^−/−^* mice to *Lck-Gfi1* transgenic mice [57] to re-express Gfi1 at the height of Notch1 target gene expression in the thymus [57], [58], [59]. Transgenic *Lck-Gfi1* expression failed to rescue germline *Gfi1^−/−^* defects in total thymocyte (Figure 4E) or ETP numbers (Figure 4F). These data corroborate that Gfi1 is required early during lymphoid progenitor development and further suggest that Gfi1 is required to properly respond to endogenous levels of Notch1 signaling.

*Gfi1^−/−^* lymphoid progenitors are reduced in number, but also fail to induce genes normally downstream of Notch signals. To delineate a requirement for Gfi1 to integrate Notch signaling versus to survive an apoptotic selection event, we attempted to rescue the loss of ETPs and total thymocytes in *Gfi1^−/−^* mice by crossing them with Bcl2-transgenic mice (*H2^K^-Bcl2*), which would block apoptosis. Although Bcl2 overexpression was able to rescue most of the Gfi1 loss-of-function phenotypes in T-ALL [37], neither total thymocyte numbers or ETP numbers returned to *Gfi1^+/+^* levels in Bcl2 transgenic *Gfi1^−/−^* mice (Figure 4E–F). Thus, forced expression of an anti-apoptotic molecule is insufficient to rescue *Gfi1* ^−/−^ T cell development defects.

## Discussion

Notch1 is a central mediator of both T cell leukemogenesis and T cell development. ICN-target genes such as *Myc* [60], [61], [62], *Hes1* [47], [63], *Notch3* [64], [65] and *IGF1R* [66] are critical to T cell development and T-ALL, and Notch signaling directly controls expression of T-cell-lineage specific identity genes such as *Tcf7* [67], [68] and *Bcl11b* [69]. Not surprisingly, interfering with the expression of Notch1 target genes disrupts Notch1 programing of developing T- or T-ALL cells. In contrast, we previously showed that Notch signaling does not directly regulate Gfi1 expression [37]. However, in this study we demonstrate that Gfi1 is still required to execute Notch1-driven developmental and pre-leukemic programs even though it is unlikely to be an ICN-downstream-target gene.

Previously, regulation of apoptosis was considered the dominant function of Gfi1 in developing T cells [9], [70]. In transformed lymphoid cells, loss of Gfi1 leads to induction of apoptosis through the exaggeration of p53-dependent target gene activation. Overexpression of Bcl2 or knockdown of p53 rescues Gfi1 loss of function phenotypes in T-ALL [37]. However, neither loss of p53 or overexpression of Bcl2 alters *Gfi1^−/−^* total thymocyte numbers (Figure 4 and data not shown). This may be due a lower threshold of DNA damage present in untransformed lymphoid precursors that is increased in T-ALL (due to oncogenic stress) resulting in hyperactivation of p53. Thus, a lack of Notch1-regulated gene expression observed in *Gfi1^−/−^* lymphoid precursors might previously have been ascribed to a selective event causing those cells that express lymphoid genes to die. Because loss of Gfi1 debilitates ICN-mediated lymphoid priming in a cell autonomous manner, we now conclude that repression of pro-apoptotic genes is only one of many biological functions that are integrated by Gfi1 during lymphoid priming and T lymphopoiesis. As expression of an anti-apoptotic effector was insufficient to rescue all of the defects associated with Gfi1 deficiency, we further conclude that Gfi1 is an obligate instructive factor that is critical to effectively maintain Notch1-dependent transcriptional programs necessary for lymphoid lineage commitment.

Previous studies have implicated Gfi1 at multiple stages of lymphoid development. For example, Gfi1 overexpression has been shown to partially rescue *Lyl1* deficiency in LMPP [71]. Moreover, Gfi1 acts downstream of Ikaros in MPPs to mediate the differentiation choice between B cells and myeloid cells [72] by antagonizing Pu.1. Given previous data demonstrating that Pu.1 can restrain Notch1 signaling in pre-T cells [73] and that Pu.1 is a *bona fide* Gfi1 target gene [72], it is attractive to hypothesize that loss of Gfi1 leads to derepression of Pu.1 which in turn opposes Notch1. However, Notch1-signaled cells appear to have alternative mechanisms to antagonize Pu.1-responsive transcriptional circuits based on the observation that upregulation of Pu.1 or Nab2 (as seen in *Gfi1^−/−^* MPP [72], [74]) in Notch-activated Gfi1 deficient cells (GSE41162) was not observed. Instead, we find that Gfi1 is required to maintain critical lymphoid transcriptional programs activated by Notch1 such as *Rag1*, *Dtx1*, and *Tcf7*. It remains unclear how Gfi1 might maintain the activation of these genes; whether Gfi1 represses other transcriptional repressors, microRNAs, or whether loss of Gfi1 leads to alternative differentiation pathways for Notch-signaled cells has yet to be elucidated.

We discovered that the phenotypes associated with Notch1 activation and Gfi1 loss of function were most severe in early lymphoid precursors, while immature SP and peripheral T cells showed modest effects. This stage-specific phenomenon could be due to the fact that Notch1 and Gfi1 are both endogenously expressed and required for the normal development of T cells from lymphoid progenitors up to TCRβ selection [9], [15], [57]. Alternatively, once T cells have completed critical development checkpoints they may no longer be susceptible to manipulation of developmental transcriptional networks. For instance, during stages of development where activation of lymphoid-associated genes is critical to establishing a T-lineage identity, Gfi1 appears to be required to maintain the activation of Notch-driven lymphoid-restricted genes such as *Tcf1*. However, in a mature T cell, either the expression of these genes is maintained by other transcription networks, or an inability to maintain their expression does not result in phenotypic consequences because the cell's developmental potential has already been achieved. In either case, our work has uncovered an epistatic relationship between Notch1 and Gfi1 that is essential for proper lymphoid development.

Loss of *Gfi1* phenocopies the loss of *Notch1* and *Tcf7* (Tcf1) with regard to the formation of ETPs, but unlike *Notch1* and *Tcf7*, *Gfi1* is also required for the survival or formation of lymphoid-primed progenitors upstream of the ETP [23], [67]. This suggests a unique role for Gfi1 in bridging lymphoid transcriptional programs from the earliest lymphoid-primed bone marrow progenitor to the thymic ETP before Notch1-regulated transcriptional programs become the dominant mechanism through which T lineage fate is enforced. Although our data do not exclude the possibility that Gfi1 participates in a shared, undiscovered, transcriptional network with other key “T cell-specific” transcription factors, it appears more likely that the phenocopy of *Gfi1^−/−^* ETP is due to the inability of Gfi1 deficient cells to integrate lymphoid progenitor transcriptional circuits, in particular those initiated by *Notch1*.

We have recently shown that Gfi1 deficient mice are protected from Notch1 mediated malignant transformation [37]. Here, we have uncovered a requirement for Gfi1 in Notch1 activated cells with implications for both normal lymphopoiesis as well as T cell transformation. Specifically, Gfi1 is required to maintain cellularity in Notch-signaled cells in a temporally regulated manner. These data help to clarify the almost absolute requirement for Gfi1 in Notch-mediated transformation. Gfi1 is required to maintain the pool of premalignant cells available for transformation, and to maintain Notch target genes essential for leukemogenesis. Thus, our data provide additional insight into the multiple mechanisms by which transcriptional networks may have evolved to protect developing lymphoid cells from transformation.

## Materials and Methods

### Mice

*LckCre*, *CD4Cre* [38], *distal LckCre* [45], *Rosa26-lox-stop-lox-Notch^IC^* [39], *Lck-Gfi1* [75], *Vav-Gfi1* [56], *Gfi1^fex4–5^* [10], *Gfi1^Δex2–3^* [76], *Gfi1 ^Δex2–5^* [6], *RosaCreER^T2^ Gfi1^fex4–5^* [46] transgenic mice have all previously been described. *Gfi1^fex4–5^* were bred to *Gfi1^Δex2–3^* mice to generate *Gfi1^fex4–5, Δex2–3^* mice to allow for more efficient deletion of the remaining floxed allele by *LckCre*, *CD4Cre* or *distal LckCre* transgenic mice. All mice were bred and housed in a specific-pathogen-free barrier facility at Cincinnati Children's Hospital Medical Center (CCHMC) Veterinary Services or at the Institut de recherches cliniques de Montréal (IRCM).

### Ethics statement

The Institutional Animal Care and Use Committee at CCHMC and the Animal Care Committee at the IRCM reviewed and approved all animal experimentation protocols, certified animal technicians, regularly observed mice in all studies and took steps to maintain animal welfare and prevent undue suffering under protocol numbers 1D09075 and 2009-12 respectively.

### Flow cytometry & FACS sorting

Thymi were harvested in Medium 199 (Invitrogen) and single cell suspensions were created. Bone marrow was flushed from femurs and tibias using Medium 199, spun down and RBC were lysed using ACK lysis buffer (Gibco). Cell counts were determined using a Coulter Counter (Beckman) and cells were then stained with various cocktails of monoclonal antibodies to the following antigens: Fc-block (2.4G2), CD4 (RM4–5), CD8a (53-6.7), CD44 (IM7), CD25 (PC61), cKit (2B8), Sca1 (D7), Flt3 (A2F10). Lineage cocktails for T cell development contained B220 (RA3-6B2), CD11b (M1/70), CD11c (N418), NK1.1, TCRγδ (UC7-13D5) and Ter119. Lineage cocktails for ETPs FACS plots contained B220 (RA3-6B2), CD3ε (145-2C11), CD8 (53-6.7), CD11b (M1/70), CD11c (N418), DX5, Gr1 (RB6-8C5), NK1.1, TCR γδ (UC7-13D5) and Ter119. Cells were stained at 4°C for 30 minutes before being washed and resuspended in PBS containing 2% FBS and 1 mM EDTA. Data was acquired on the BD LSRII, LSRFortessa or FACSCanto. Cells were FACS sorted on the BD FACSAriaII and recovered in PBS with 50% FBS.

### PCR and gene expression analyses

Polymerase chain reaction (PCR) detection of the *Gfi1^fex4–5^* allele was performed with primers 5′-CAGTCCGTGACCCTCCAGCAT-3′ and 5′-CTGGGAGTGCACTGCCTTGTGTT-3′, whereas detection of the *Gfi1* ^Δ*ex4–5*^ allele was performed with primers 5′-CAGTCCGTGACCCTCCAGCAT-3′ and 5′-CCATCTCTCCTTGTGCTTAAGAT-3′. Gene expression analysis was performed on RNA isolated from TRI Reagent (Sigma) by phenol-chloroform extraction or by the RNeasy kit (QIAGEN). cDNA was synthesized from purified RNA using the cDNA High Capacity Archive Kit (Applied Biosystems) according to the manufacturer's instructions. Gene expression was assessed using Taqman probes (Applied Biosystems) or primers for *cMyc* (Mm03053277_s1, Mm00487803_m1), *Dtx1* (Mm00492297_m1), *Hes1* (Mm00468601_m1, Mm01342805_m1), *Hey1* (Mm00468865_m1), *Ptcra* (Mn00478361_m1), *Ccnd1*(Mn00432359_m1), *Notch1* (Mm00435245_m1, Mm00435249_m1), *Notch3* (Mm01345646_m1) on an ABI Prism 7900. Threshold values were calculated and normalized to the endogenous control, *Gapdh* (Mm99999915_g1); then, the ΔΔCT method was used to calculate the fold change compared to *Gfi1^+/+^* controls. Gene array data (GSE20282 or GSE41162) was analyzed using GeneSpring (version 12.0 Agilent Technologies) or the R software package.

### Cell culture and in vitro differentiation

OP9-DL1 cells were cultured in 24-well plates at a concentration of 2×10^4^cells/ml in α-MEM media supplemented with 20% FBS (charcoal stripped), β-mercapto ethanol, sodium pyruvate, and non-essential amino acids. OP9-DL1 cells were seeded 24 h before FACS sorted ETP were directly sorted onto the monolayer. Fresh IL7 (1 ng/mL) and Flt3L (5 ng/mL) were then added. Media was changed every 4–5 days and developing T cells were transferred onto a new monolayer of OP9-DL1 cells with fresh media and cytokines.

### Retroviral transduction and CFU assays

Lineage negative cells were isolated from total BM using magnetic separation (Miltenyi) and then placed into StemSpan SF media (StemCell Technologies) containing IL-3, IL-6, IL-7, SCF, Flt3L and human IL-11 (Miltenyi) with 1% Glutamine and 1% Pen/Strep (Gibco). Cells were expanded for two days before being placed on Retronectin (Takara) coated plates preloaded with viral supernatants harvested from MigR1-ICN-ires-eGFP transfected 293T cells. Viral supernatants were spinfected at 1000 g at 4°C for 30 minutes. The process was repeated twice and the cells were expanded for 48 hours before FACS-sorting. eGFP+ cells were resuspended in MethoCult semi-solid media (StemCell Technologies) and allowed to grow for one week. CFU were enumerated, cells were then dissociated, counted and replated. 4-OHT was added at a final concentration of 1 µM to induce Cre activity. *Gfi1* deletion was confirmed by PCR; any CFU demonstrating incomplete excision of floxed *Gfi1* was excluded from gene expression array analysis.

## Supporting Information

Figure S1 Temporal deletion of Gfi1 phenocopies *Gfi1^−/−^* T cell development. (A) Top: Schematic of T cell development demonstrating that the proximal Lck-driven Cre activity is off during early stages (DN1–2), but activates later during the DN3 stage of T cell development. Bottom: Schematic of the floxed *Gfi1* locus. (B) Total thymocyte numbers from Gfi1*^fex4–5/Δex2–3^* (n = 2) and *LckCre^+^ Gfi1^fex4–5/Δex2–3^* (n = 3) mice (**p<0.01). (C) PCR analysis of 3 separate mice for floxed (top, *Gfi1^fex4–5^*) and deleted (bottom, *Gfi1^Δex4–5^*) alleles of *Gfi1*. Tail samples from the same mouse serve as a Cre negative tissue control. (D) Total cell numbers of CD4^+^ and CD8^+^ single positive thymocytes from indicated genotypes. (E) Ratio of CD4 to CD8 thymocytes in *Gfi1^fex4–5/Δex2–3^* vs. *LckCre^+^Gfi1^fex4–5/Δex2–3^* mice. *p≤0.05, **p≤0.01.
(TIF)

Figure S2 Distal Lck Cre (DLCre) expression is limited in thymocytes. (A) Top: Schematic of T cell development demonstrating that the distal Lck-driven Cre activity is off during early stages (DN1–4), but activates later during the transition out of the DP stage of T cell development. Bottom: Schematic of the ICN transgene marked by eGFP and endogenous *Gfi1* locus. (B) Representative FACS plots of GFP negative or positive live-gated thymocytes demonstrating DLCre transgenic expression is limited in the thymus and primarily observed in CD8+ and DP thymocytes.
(TIF)

Table S1 A subset of significantly deregulated genes between ICN-transduced *Gfi1^f/f^* and *Gfi1^Δ/Δ^* CFU with a p-value less than 0.05 are displayed in the same order as in the heatmap in Figure 3D (cluster order). All values represent the Log_2_ of the normalized gene expression values calculated with GeneSpring software for each of the indicated genes. FACS-sorted lymphoid progenitor microarrays were reanalyzed from a recent publication [49]. The values in this table were used to generate the PCA in Figure 3C. Hematopoietic stem cell (HSC), lymphoid-primed multipotent progenitor (LMPP), early T lineage progenitor (ETP), common lymphoid progenitor (CLP), pro-B lymphocyte (ProB), double negative (CD4−, CD8−, DN).
(XLSX)

Table S2 All genes that demonstrated a +/−2 fold change between ICN-transduced *Gfi1^f/f^* and *Gfi1^Δ/Δ^* CFU were examined for enrichment of Gene Ontology (GO) Cellular Components. The “Cell Surface” (GO:0009986) was the most significantly enriched term with a p-value of 2.49×10^−16^. The gene names, gene symbols and fold change between ICN-transduced *Gfi1^f/f^* and *Gfi1^Δ/Δ^* CFU are shown sorted by fold change. The color-coded key demonstrates the level deregulation in ICN-transduced *Gfi1^Δ/Δ^* CFU.
(XLSX)

Table S3 All genes that demonstrated a +/−2 fold change between ICN-transduced *Gfi1^f/f^* and *Gfi1^Δ/Δ^* CFU were examined for enrichment of Gene Families. The “CD Molecule” gene family was the most significantly enriched term with a p-value of 3.4×10^−30^. The gene names, gene symbols and fold change between ICN-transduced *Gfi1^f/f^* and *Gfi1^Δ/Δ^* CFU are shown sorted by fold change. The color-coded key demonstrates the level deregulation in ICN-transduced *Gfi1^Δ/Δ^* CFU.
(XLSX)
