# Supplementary figures and images for: Growth factor independent-1 Maintains Notch1-Dependent Transcriptional Programming of Lymphoid Precursors
Source: PLoS Genet. 2013 Sep 12;9(9):e1003713. doi: 10.1371/journal.pgen.1003713 (PMC3772063; doi:10.1371/journal.pgen.1003713)

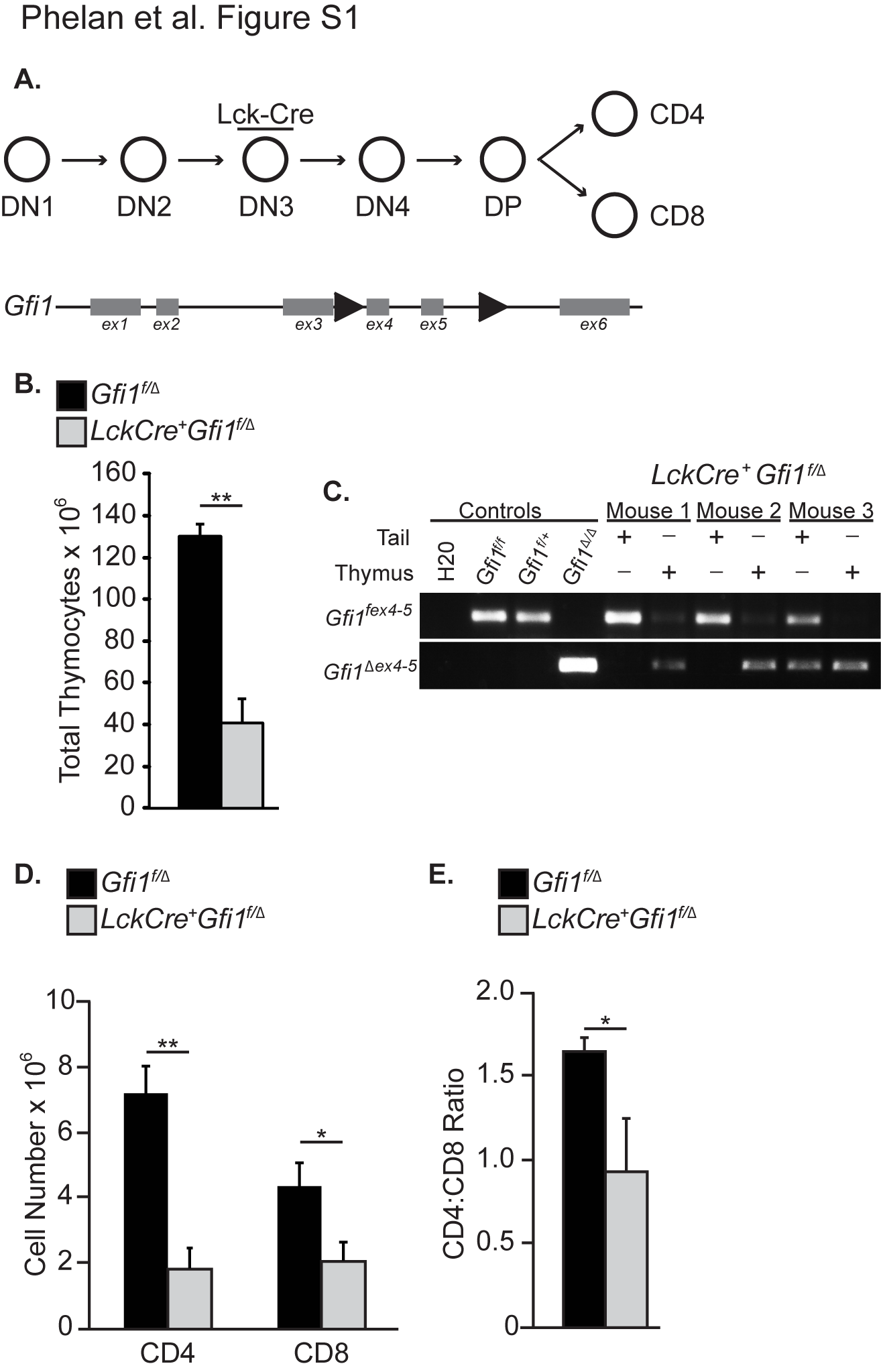

Supplement: Figure S1 — Temporal deletion of Gfi1 phenocopies Gfi1−/− T cell development. (A) Top: Schematic of T cell development demonstrating that the proximal Lck-driven Cre activity is off during early stages (DN1–2), but activates later during the DN3 stage of T cell development. Bottom: Schematic of the floxed Gfi1 locus. (B) Total thymocyte numbers from Gfi1fex4–5/Δex2–3 (n = 2) and LckCre+ Gfi1fex4–5/Δex2–3 (n = 3) mice (**p<0.01). (C) PCR analysis of 3 separate mice for floxed (top, Gfi1fex4–5) and deleted (bottom, Gfi1Δex4–5) alleles of Gfi1. Tail samples from the same mouse serve as a Cre negative tissue control. (D) Total cell numbers of CD4+ and CD8+ single positive thymocytes from indicated genotypes. (E) Ratio of CD4 to CD8 thymocytes in Gfi1fex4–5/Δex2–3 vs. LckCre+Gfi1fex4–5/Δex2–3 mice. *p≤0.05, **p≤0.01. (TIF) [file pgen.1003713.s001.tif]

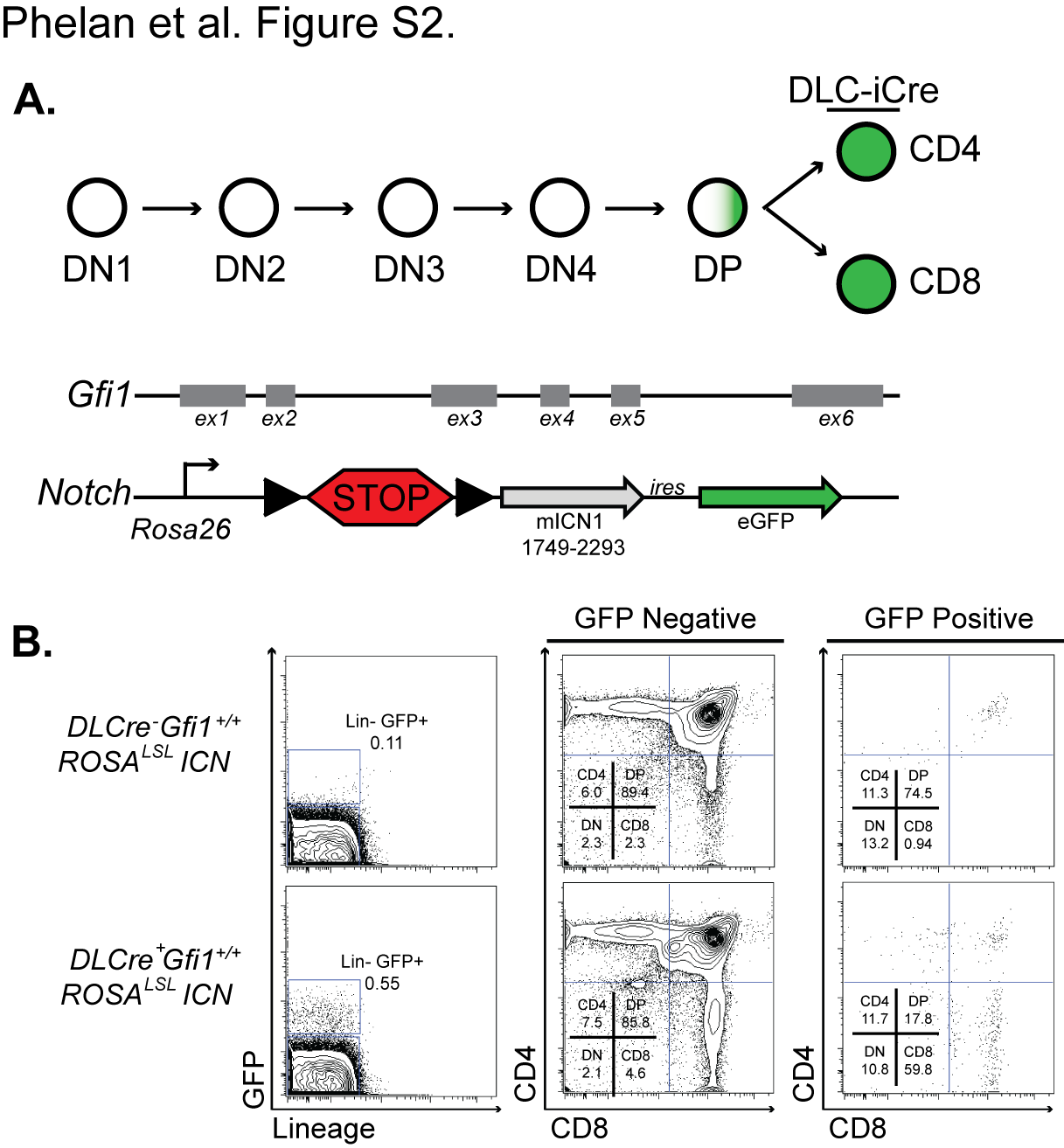

Supplement: Figure S2 — Distal Lck Cre (DLCre) expression is limited in thymocytes. (A) Top: Schematic of T cell development demonstrating that the distal Lck-driven Cre activity is off during early stages (DN1–4), but activates later during the transition out of the DP stage of T cell development. Bottom: Schematic of the ICN transgene marked by eGFP and endogenous Gfi1 locus. (B) Representative FACS plots of GFP negative or positive live-gated thymocytes demonstrating DLCre transgenic expression is limited in the thymus and primarily observed in CD8+ and DP thymocytes. (TIF) [file pgen.1003713.s002.tif]
